# Prevention of Ulcerative Colitis by Autologous Metabolite Transfer from Colitogenic Microbiota Treated with Lipid Nanoparticles Encapsulating an Anti-Inflammatory Drug Candidate

**DOI:** 10.3390/pharmaceutics14061233

**Published:** 2022-06-10

**Authors:** Chunhua Yang, Junsik Sung, Dingpei Long, Zahra Alghoul, Didier Merlin

**Affiliations:** 1 Digestive Disease Research Group, Institute for Biomedical Sciences, Georgia State University, Atlanta, GA 30303, USA; jsung9@gsu.edu (J.S.); dlong26@gsu.edu (D.L.); zalghoul@gsu.edu (Z.A.); dmerlin@gsu.edu (D.M.); 2 Atlanta Veterans Affairs Medical Center, Decatur, GA 30302, USA; 3 Department of Chemistry, Georgia State University, Atlanta, GA 30303, USA

**Keywords:** natural-lipid nanoparticles, M13, ex vivo culture, microbiota-secreted metabolites, IL-10 knockout, chronic inflammation

## Abstract

Modulating the gut microbiota composition is a potent approach to treat various chronic diseases, including obesity, metabolic syndrome, and ulcerative colitis (UC). However, the current methods, such as fecal microbiota transplantation, carry a risk of serious infections due to the transmission of multi-drug-resistant organisms. Here, we developed an organism-free strategy in which the gut microbiota is modulated ex vivo and microbiota-secreted metabolites are transferred back to the host. Using feces collected from the interleukin-10 (IL-10) knockout mouse model of chronic UC, we found that a drug candidate (M13)-loaded natural-lipid nanoparticle (M13/nLNP) modified the composition of the ex vivo-cultured inflamed gut microbiota and its secreted metabolites. Principal coordinate analysis (PCoA) showed that M13/nLNP shifted the inflamed microbiota composition toward the non-inflamed direction. This compositional modification induced significant changes in the chemical profiles of secreted metabolites, which proved to be anti-inflammatory against in vitro-cultured NF-κβ reporter cells. Further, when these metabolites were orally administered to mice, they established strong protection against the formation of chronic inflammation. Our study demonstrates that ex vivo modulation of microbiota using M13/nLNP effectively reshaped the microbial secreted metabolites and that oral transfer of these metabolites might be an effective and safe therapeutic approach for preventing chronic UC.

## 1. Introduction

Ulcerative colitis (UC) is a form of chronic inflammatory bowel disease (IBD) that affects more than five million patients worldwide [1]. Pathophysiological studies have indicated that UC development is a long-term process that includes various unclear etiologies. Accumulating evidence has shown that dysbiosis (irregular gut microbiota composition) is associated with the progression of UC [2]. Recently developed fecal microbiota transplantation (FMT) technologies have offered promising new approaches for modulating dysbiosis to treat UC [3,4]. However, FMT requires a prolonged treatment time and may cause many side-effects, including severe exogenous infections [5]. Thus, establishing a safer and more efficient technique is an unmet need in the clinical treatment of UC.

The interleukin-10 knockout (IL10 KO) mouse model is a chronic inflammation model that closely replicates the disease features of human UC [6,7]. Unlike the dextran sulfate sodium (DSS)-induced acute colitis model, which exhibits a significant increase in the permeability of the mucus layer, which enables the enhanced permeability (EPR) effect to be targeted for drug delivery, the mucus layer of IL10 KO mice is not drastically compromised. The development of UC in IL-10 KO mice is a long process that lasts more than 10 weeks [8]. Our previous studies indicated that 6-week-old IL-10 KO mice were still in the healthy (non-inflammatory colon) stage, whereas their 10- to 12-week-old counterparts exhibited a mild inflammation that developed gradually and was reflected by a slow increase in the fecal lipocalin-2 (LCN-2) concentration [8].

Recent studies have shown that 6-shogaol-loaded natural lipid nanoparticles (6S/nLNP) can quickly regulate the gut microbiota composition, trigger fecal metabolic change, and inhibit inflammation in the DSS-induced acute colitis model [9]. Such studies suggested that nLNP offer an excellent drug-delivery system that can accelerate the microbiota-modulating effects of 6-shogaol. When 6S/nLNP was orally administered, transferases rapidly converted 6-shogaol to its phase-II metabolite (glutathione-conjugated), M13, which was later found to promote colonic wound healing and reduce inflammation in an acute colitis model [10]. M13 also demonstrates improved drug-like characteristics, such as increased water solubility and reduced cytotoxicity, compared to 6-shogaol. The efficacy of M13 may involve an ability to modulate the gut microbiota composition and consequent microbiota-secreted metabolites (indirect effect), as well as an ability to regulate the host colonic immune cells (direct effect).

To further study whether modulation of the microbiota composition is the single most important factor in preventing disease progression, we collected the feces from 6-week-old non-inflamed and 10-week-old inflamed IL-10 KO mice and cultured the 10-week-old fecal microbiota with M13/nLNP-containing medium in an anaerobic environment. In parallel, we cultured the 10-week-old fecal microbiota with PBS, free M13, or empty nLNP-containing medium, and 6-week-old fecal microbiota with PBS-containing medium. Interestingly, the M13/nLNP group presented the most significant changes in the microbiota composition, which was reshaped towards the non-inflamed stage (the composition found in cultured 6-week-old fecal microbiota). This exciting discovery encouraged us to investigate the changes in microbiota-secreted metabolites with the goal of developing an organism-free strategy to avoid the pathogen-transfer risk of FMT.

Using ex vivo culture-derived microbial metabolites (rather than the microbiota itself) offers an excellent opportunity to treat disease without the risk of severe exogenous infections associated with FMT [11]. Our in vitro studies showed that M13/nLNP-related microbial metabolites could inhibit the TNF-α activation step of NF-κβ signal transduction. We hypothesized that M13/nLNP-reshaped microbial metabolites could impact the progress of UC and tested whether feeding IL-10 KO mice with their ex vivo M13/nLNP-altered microbiota-secreted metabolites could prevent the development of chronic UC between 11 and 13 weeks.

## 2. Materials and Methods

### 2.1. Chemicals

L-Glutathione reduced, potassium chloride (KCl), ethanol (200 proof), methanol, dichloromethane, and acetonitrile (LC-MS grade) were purchased from Sigma-Aldrich (St. Louis, MO, USA). The 6-shogaol (98% purity) was purchased from Chengdu alfa biotechnology (Chengdu, China). Formic acid (98+%, LC-MS grade), phosphate-buffered saline (Corning^™^ PBS, 1×, Corning, NY, USA), and Dil Stain (1,1′-Dioctadecyl-3,3,3′,3′-Tetramethylindocarbocyanine Perchlorate (DiIC18(3))) were purchased from Fisher Scientific (Hampton, NH, USA). Fetal bovine serum (FBS) was purchased from R&D Systems (Flowery Branch, GA, USA). Ultrapure deionized water was obtained using a Milli-Q water system (Millipore, Bedford, MA, USA). Dextran sulfate sodium (DSS, 36–50 kDa) was obtained from MP Biomedicals (Santa Ana, CA, USA). M13 was synthesized and purified using the method published previously [12].

### 2.2. Preparation of M13/nLNP

#### 2.2.1. Lipid Extraction

Fresh ginger (*Zingiber officinale* Rosc.) roots (~2.5 kg) were bought from the Buford Highway farmers’ market (Doraville, GA, USA). Ginger-derived nanoparticles band 2 (GDNPs-2; ~200 mg) was isolated using a gradient ultracentrifugation method [13,14]. We extracted the lipids from GDNPs-2 using a modified liquid–liquid extraction (LLE) method introduced by Bligh and Dyer [15,16]. First, 15 mL of GDNPs-2 suspension (1 mg/mL in PBS) was added into a glass separatory funnel, then 60 mL of methanol/dichloromethane (2:1; *v*/*v*) was added, and the funnel was gently shaken to mix the liquid. After mixing, we sequentially added dichloromethane (20 mL) and ddH_2_O (20 mL) into the funnel. The mixture was then shaken thoroughly at room temperature (RT) 10–15 times, and we opened the stopcock with the funnel upside down to release the pressure. After standing in the funnel for 15 min, the bottom phase (organic phase) was separated and transferred into a new glass separatory funnel. Then, the sample was washed with 5 mL KCl solution (1 mol/L) and then with 5 mL water. Finally, the organic phase was dried within a vacuum rotavapor between 40–45 °C and stored at −20 °C.

#### 2.2.2. Preparation of M13/nLNP

Next, 2 mL of extracted lipids (5.0 mg/mL in dichloromethane) was added to a 500 mL pear-shaped recovery flask and dried under reduced pressure to form a thin lipids film. Then, 50 mL M13 solution (0.1 mg/mL in PBS) was added to the flask and mixed with the dried lipids film. The mixture was then subjected to bath sonication (55 °C) for 5 min with proper pipetting (50~100 times). After the mixture formed a homogenized suspension, another 50 mL of prewarmed PBS buffer (55 °C) was added, then the mixture was sonicated for another 5 min. Next, the M13/nLNP suspension was maintained at 55 °C and passed through a NanoSizer^™^ liposome extruder equipped with a 200 nm polycarbonate membrane (T&T Scientific Corporation, Knoxville, TN, USA) 20–25 times. Empty nLNP was made by the same protocol described above without adding M13. Finally, the M13/nLNP or empty nLNP suspensions were submitted to ultracentrifuge (at ~120,000× *g* for 45 min), and the nanoparticles were retrieved by dispersing the pellet in deionized water or PBS with repeated pipetting in the bath sonication.

#### 2.2.3. Characterization and Drug Release of the M13-Loaded Lipid Nanoparticles

To characterize the M13/nLNP or nLNP under physiologically relevant conditions, we suspended nanoparticles in PBS at a proper concentration (between 10 to 100 µg/mL). Then, 0.8 mL of the suspension was added to the Zetasizer cuvet, and particle diameter (nm) and zeta potential (mV) of M13/nLNP or nLNP were measured by the dynamic light scattering (DLS) method using Malvern Zetasizer Nano ZS90 Apparatus (Malvern Instruments, Worcestershire, UK) at RT. We also characterized the zeta potential of M13/nLNP or nLNP in pure water by suspending the M13/nLNP or nLNP in deionized water. The average and standard deviations of the diameters (nm) or zeta potentials (mV) were calculated using three runs. The morphological image was acquired by CoreAFM atomic force microscopy (Nanosurf, Liestel, Switzerland). In general, ~2.5 µL of 1–5 µg/mL M13/nLNP or nLNP suspension (diluted in purified water) were dropped on freshly peeled mica sheets and dried at RT for at least 2 h. Then, the M13/nLNP- or nLNP-loaded mica sheets were submitted to the AFM scan stage. The scanning mode was set as a dynamic force, with a scan area of (10 × 10 µm^2^) and a power gain of 2500 at the z-axis.

The loading efficiencies (LE) of M13 to nLNP were tested using the centrifugation method with Amicon^®^ Ultra-4 centrifugal filters (Ultracel^®^—100K). First, M13/nLNP was suspended in 2mL PBS (n = 3) and added to the apical side of the Amicon^®^ filters, and the filters were immediately centrifuged at 1000× *g* for 10 min. Then, the passing-through solutions (~0.5 mL, containing free M13) or the total M13/nLNP solutions (0.5 mL) were mixed with 1.0 mL acetonitrile and vortexed for 1 min. Finally, the vortexed solutions were passed through 0.22 μm nylon filters and transferred to HPLC vials for analysis.

Slide-A-Lyzer mini dialysis cup (10 K molecular weight cutoff (MWCO), 15 mL) was used to test the drug-release profile of M13/nLNP at 37 °C. The dialysis cup was stabilized by adding 0.5 mL of culture medium (pH 7.4) to the apical side of the dialysis cup. The basal side of the cup was filled with a 12.5 mL culture medium. All the mini dialysis cups were stabilized at 37 °C for ~0.5 h before use. Then, 0.5 mL of M13/nLNP (0.5 mg/mL) was added to the apical side of the tube. The samples were shaken at 200 rpm at RT, and 0.5 mL suspension was taken out, respectively, from the apical and basal sites of the dialysis cups after different time-points. All samples were mixed with the 1.0 mL of acetonitrile, shaken, and passed through a 0.22 μm nylon filter before injection.

High-performance liquid chromatography (HPLC) analysis was performed to evaluate the LE or drug release using an Agilent^®^ 1100 LC System (Agilent Technologies Inc.; Santa Clara, CA, USA). A Zobax C18 column from Agilent (2.1 × 50 mm, 5 µm, 80 A) was used for the separation, and the UV-detection wavelength was set at 282 nm. The drug LE was calculated using the formula: ((total drug − free drug)/total drug) × 100%. An M13 PBS solution (0.5mg/mL) was used to determine the absorption of free M13 on the Amicon^®^ Ultra-4 centrifugal device by comparing the HPLC peak area of the M13 before and after filtration. The 5.5% of absorption (average, n = 3) of free M13 on the filtration device was used to adjust the free drug concentration in the formula that calculated the LE. The drug release was calculated using the formula: (free drug at basal side/total drug) × 100%. Similarly, 0.5 mg/mL free M13 solution was used to determine the absorption of free M13 on the dialysis device and the observed 20.6% of absorption (average, n = 3) of free M13 on the device was used to adjust the basal side drug concentration in the formula that calculated the drug release of M13/nLNP. The obtained drug-release data (released%-time) were input into Windows Excel (2013) to calculate the modeling parameters and R^2^ value, and the drug-release kinetics and modeling were visualized by Graphpad Prism (Version 8.3.1).

### 2.3. Ex Vivo Culturing of the Fecal Microbiota

Mice (female, 3 weeks old, strain: B6. 129P2-IL10 tm1Cgn) were purchased from the Jackson Laboratory and housed in the animal facility of Georgia State University (GSU). The animal procedures in this study were approved by the University Committee on Use and Care Animals at GSU (IACUC, Protocol # A20039).

We collected two fecal pellets from each IL-10 KO female mouse when she was 6 weeks old and 10 weeks old (n = 4). The feces was put into EP tubes and immediately covered with 1 mL of PBS/Glycol solution (30% Glycol) and stored at −80 °C. Before ex vivo culture, all culture tubes (75 mL) were filled with 25 mL autoclaved culture medium spiked with 0.25 mL 100 µg/mL of free M13, empty nLNP, M13/nLNP, or 0.25 mL 1xPBS. Then, the culture tubes were flushed 10 times and filled with premixed gas of Nitrogen (80%) and carbon dioxide (20%) and sealed with an airtight rubber stopper; continuous premixed gas was offered to the tubes via prefilled gas balloon (1000 mL). The fecal suspension was homogenized for 10 min and went through the cell strainer (Falcon^®^, 40 µm, Nylon) in the anaerobic chamber to remove the fibers. The filtrated suspension was centrifuged at 1000× *g* for 5 min to obtain the gut microbiota pellets, then the microbiota pellets were resuspended in the sterile PBS and 100 µL of the suspension was spiked (with syringe needles) immediately into the prefilled 75 mL tubes. All the tubes were cultured under the anaerobic condition at 37 °C for 24 h. After the culture, the UV absorption (at 600 nm) of the cultured suspension was measured, and 1mL suspension was centrifuged at 1000× g to obtain the microbiota pellets for compositional analysis using the WGS technique.

### 2.4. Microbiota Composition Analysis

After the ex vivo culture, the UV absorption of the suspension was measured at 600 nm by Pharmacia Biotech U.V./Visible Spectrophotometer (Ultrospec 1000), and 1 mL of the suspension (~1.00 absorbance unit) was centrifuged at 1000× *g* to get the pellet. Then, after removing the supernatant, the pellet was immediately suspended in the Transnetyx microbiome sample collection tube, and all the samples were submitted to the Transnetyx automatic whole-genome sequencing (WGS) platform. The platform employed shallow shotgun WGS with a minimum read depth of two million paired-end reads for the methodology and provides species/substrain-level taxonomic resolution on each sample. The analysis includes all microbe types, including bacteria, viruses, fungi, protists, and archaea. Data visualization of taxonomy, alpha- and beta-diversity, clustering, and PCoA were performed at the Onecodex (https://www.onecodex.com, accessed on 15 December 2021) cloud-computing website.

### 2.5. LC-MS Analysis of Microbiota-Secreted Metabolites

#### 2.5.1. Sample Preparation

First, 1 mL of ex vivo cultured fecal microbiota suspension was centrifuged at 1000× *g* at 4 °C for 5 min, and the supernatants (0.75 mL) were collected and filtered through the 0.22 µm spin filter at 10,000 rpm for 10 min. Then, the filtrated solution was freeze-dried by a lyophilizer (Labconco, Kansas City, MO, USA) for 24 h and stored at −20 °C. To prepare the LC-MS samples, we added 0.75 mL of 70% icy cold methanol to reconstitute the samples. All samples were then vortexed for ~2 min and centrifuged at >12,000 rpm at 4 °C for 10 min. We centrifugated all the samples using the membrane filter (molecular weight cutoff [MWCO]: 3500 Dalton) to remove the macromolecules and then transferred them to 2.0 mL HPLC injection vials for the LC-MS analysis. LC-MS quality control (QC) samples were made by mixing 6 different samples, and HPLC retention times of QC samples were used to evaluate the stability of the LC-MS system.

#### 2.5.2. Ultra-High-Performance Liquid Chromatography-Mass Spectrometry (UPLC-MS)

High-resolution highly accurate mass spectrometry (HRMS) data were acquired by UPLC-MS, equipped with an Ultimate-3000 LC system and Q-Exactive mass spectrometer (Thermo Fisher Scientific, Waltham, MA, USA). The data were acquired with ESI-MS in non-targeted MS/MS mode. The UPLC system contained a Thermo Hyper gold C18 (100 × 2.1 mm, 1.9 µm) column for the peak separation. The two-phase mobile phase was 0.1% formic acid, 5% acetonitrile, 94.9% water (solvent A), and 0.1% formic acid-99.9% acetonitrile (solvent B). UPLC was run in a gradient elution mode with the flow rate set as 0.3 mL per min, the UPLC column was kept at 40 °C, and the sample injection module was kept at 4 °C.

#### 2.5.3. Metabolic Profile and Identification of Metabolites

UPLC-HRMS data were converted to the format of *.mzXML by ProteoWizard (Version: 3.0.22098, Palo Alto, CA, USA) and uploaded to the XCMS website (https://xcmsonline.scripps.edu, accessed on 15 December 2021). The uploaded HRMS data from different groups of ex vivo cultured microbiota-secreted metabolites (PBS, free M13, empty nLNP, and M13/nLNP treated) were matched via Metlin online metabolomics database (https://metlin.scripps.edu, accessed on 15 December 2021). The current Metlin database contains over a million molecules ranging from lipids, steroids, plant, and bacterial metabolites. The database also contains small peptides, carbohydrates, exogenous drugs/metabolites, central carbon metabolites, and toxicants. The search used positively charged mass (including [M+H]^+^, [M-H_2_O+H]^+^, [M+Na]^+^, and [M-H_2_O+Na]^+^), with an M/z accuracy of no more than 3.0 ppm.

### 2.6. Effect of Microbiota Secreted Metabolites on the NF-κβ Reporter Cells

NF-κβ/293/GFP-Luc^™^ cell line (Passage number 5–7, System Biosciences, Palo Alto, CA, USA) was plated at a 1 million cells/mL concentration into each well of a 24-well plate. After the cells were attached, metabolites secreted from M13/nLNP, free M13, or empty nLNP treated microbiota (normalized to 1mg/mL) were mixed with culture medium (25 μM), and were then added to the plate and incubated for 4 h. TNF-α was added to the medium (10 ng/mL final concentration) and incubated for another 18 h to activate the NF-κβ signal transduction pathway. The luminance of the reporter cells was then quantitated by Illumination™ Firefly Luciferase Enhanced Assay Kit (Gold bio) with a BioTek Synergy LX multi-mode luminometer (Agilent, Santa Clara, CA, USA).

### 2.7. Treament of IL-10 KO Mice

A mouse model of chronic colitis was established by using IL-10 KO mice (B6. 129P2-IL10 tm1Cgn, the Jackson Laboratory). Mice (female, 3–4 weeks old) were housed in the animal facility of GSU, and fecal LCN-2 concentrations were monitored weekly using the EILSA kit (R&D system, Minneapolis, MN, USA). Animal experiments were performed following GSU’s research guidelines that cover animals’ humane care and use. The procedures used in this study were approved by the University Committee on Use and Care Animals at GSU (IACUC, Protocol # A20039).

On week 10, fecal LCN-2 measurement showed that all mice had developed low-grade inflammation. The week 10 feces was then ex vivo cultured (incubated with M13/nLNP containing medium). For each dose, 2 mL of ex vivo cultured fecal microbiota suspensions (1 absorbance unit/mL) were centrifuged at 1000× *g* at 4 °C for 5 min, and the supernatants (~1.8 mL) were collected and filtered through the 0.22 µm spin filter at 10,000 rpm for 10 min. Then, the filtrated solution was freeze-dried by a lyophilizer (Labconco, Kansas City, MO, USA) for 24 h and stored at −20 °C before use. To prepare the gavage samples, we added 1 mL of 70% icy cold ethanol to reconstitute the samples. All samples were then vortexed for ~2 min and centrifuged at >12,000 rpm at 4 °C for 10 min. We centrifugated all the samples through the membrane filter (molecular weight cutoff [MWCO]: 3500 Dalton) to remove the macromolecules and then transferred them to a speed vacuum for drying. The dried samples were suspended in 0.2 mL of PBS. Each mouse (11 weeks old) was gavaged with 0.2 mL above suspension from M13/nLNP cultured groups or normal PBS daily for 14 days. After treatment, the 13-week-old mice were euthanized, and the blood and tissues were collected for analysis.

### 2.8. Fecal Lipocalin-2 (LCN-2) ELISA Assay

Two pellets of feces were placed in a 2 mL tube, lyophilized for 24 hrs, and accurately weighed. Then, 1 mL PBS (containing 0.1% Tween 20) for every 100 mg dried feces was added into the tube, the fecal samples were homogenized by vortex for 15 min, and supernatants were collected after centrifugation at 10,000× *g*, 4 °C for 15 min. Samples and standards were loaded on capture antibody precoated 96-well plates and incubated for 2 h at RT. After 3 washing steps, detection antibodies, streptavidin-HRP, and substrate solution were added. Complete aspiration of each solution and washing was performed between each procedure. Optical density was read at 450 nm and 540 nm immediately after adding the stop solution. Readings were subtracted from 450 nm to 540 nm to correct imperfect data recording.

### 2.9. Colonic Myeloperoxidase (MPO) Assay

Preweighed colon tissues (~50 mg) were washed with PBS (3 times) and homogenized in 0.5% hexadecyltrimethylammonium bromide (HTAB). Sonication was followed up at 50% amplitude, 5 s on/off. Three freezing and thawing cycles were performed at −80 °C and 37 °C conditions. Then, the samples were centrifuged at 10,000× *g* for 15 min at 4 °C. Clear supernatants and MPO standards were loaded on 96-well plates, and reactive buffers were added to each well immediately after preparing the solutions. After colors developed, optical density was read using a BioTek Synergy 2 plate reader at 450 nm.

### 2.10. Quantitative Level of Colonic Cytokine mRNA Expression

Colon tissues (~25 mg) were weighed and homogenized in 250 µL of lysis buffer (RLT buffer, Qiagen, Hilden, Germany), then 250 µL of icy cold 70% ethanol was added, and total RNAs were extracted and purified by RNeasy Mini Kit (Qiagen, Hilden, Germany). Maxima cDNA Synthesis Kit (Thermo Scientific, Waltham, MA, USA) generated complementary DNA (cDNA). After 20 times dilution, Maxima SYBR Green/ROX qPCR Master Mix (Thermo Scientific, Waltham, MA, USA) was used to analyze the RNA expression level of pro-inflammatory cytokines (TNF-α, IL-6, and IL-1b) with a 96-well format thermal cycler (Eppendorf, Hamburg, Germany). The housekeeping gene, 36B4, was used as a normalization cytokine. The following sense and anti-sense primers were used: TNF-α: 5′-AGG CTG CCC CGA CTA CGT-3′ and 5′-GAC TTT CTC CTG GTA TGA GAT AGC AAA-3′; IL-1b: 5′-TTG ACG GAC CCC AAA AGA TG-3′ and 5′-AGA AGG TGC TCA TGT CCT CAT-3′; IL-6: 5′-ACA AGT CGG AGG CTT AAT TAC ACA T-3′ and 5′-TTG CCA TTG CAC AAC TCT TTT C-3′, and 36B4: 5′-TCC AGG CTT TGG GCA TCA-3′ and 5′-CTT TAT CAG CTG CAC ATC ACT CAG A-3′. Data were recorded and analyzed by Mastercycler ep Realplex version 2.2 (Eppendorf, Hamburg, Germany).

### 2.11. H&E Staining

Sliced mouse colons were fixed for ~48 h in 10% buffered formalin solution at RT and transferred to 70% ethanol for another 48 h. Then, the colon tissues were processed and paraffin-embedded as formalin-fixed paraffin-embedded (FFPE) blocks embedded in paraffin. After deparaffinization, the FFPE blocks were sectioned at 4 µm onto positively charged SuperFrost slides for maximal tissue adherence. The colon sections (~4 µm thickness) were stained with hematoxylin/eosin (H&E) using a standard protocol [17]. The blue/purple staining represents nuclei; the red staining represents cytoplasm, muscle, and erythrocytes; the pink staining represents collagen and mitochondria; and the purplish-red staining represents basophils. Whole slide images are generated using a Pannoramic SCAN (3D Histech) in the lab of the reveal bioscience company. The range of integrated AI-powered image QC tools automatically assesses focus, tissue and slide artifacts, and image quality at scale.

### 2.12. Data, Graphic, and Statistical Analysis

Microsoft Excel 2013 was used for data recording, processing, and statistical analysis. Microsoft PowerPoint 2013 was used for graphing. GraphPad Prism 8.31 was utilized for data analysis and visualization. All the data presented are biological replicates, and the outliers were calculated and removed by the outlier calculator in GraphPad with an alpha value of 0.05. Significance was determined using unpaired two-tailed Student’s *t*-test and differences were noted as significant (* *p* < 0.05, ** *p* < 0.01, and *** *p* < 0.001).

## 3. Results

### 3.1. Characterization of M13/nLNP

**Figure 1:**
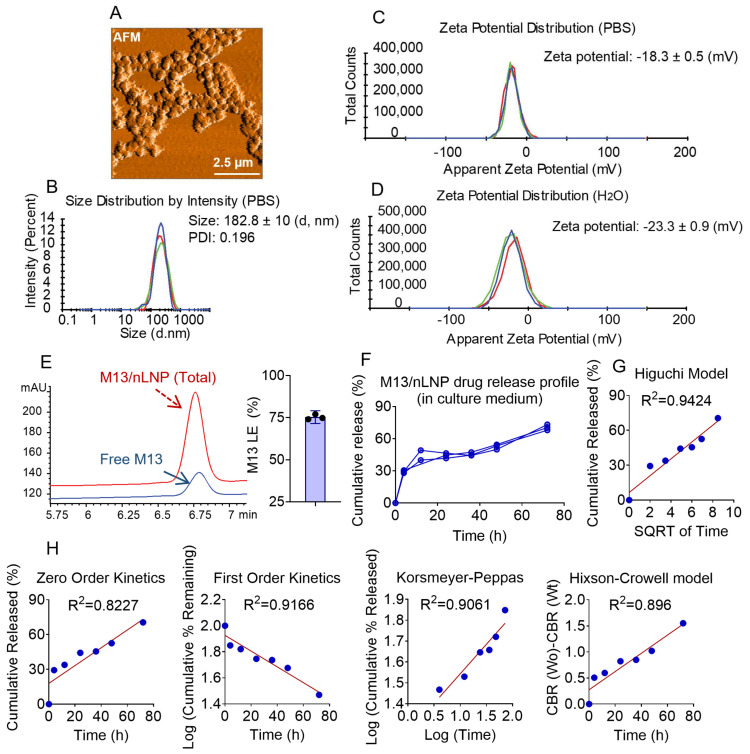
AFM, size, zeta potential, loading efficiency, and drug release of M13/nLNP. (**A**) Representative AFM image; (**B**) size distribution showed that the average size of M13/nLNP was 182.8 ± 10 nanometers (n = 3) and with a Polydispersity Index (PDI) of 0.196; (**C**) zeta potential of M13/nLNP was −18.3 ± 0.5 mV in PBS (n = 3); (**D**) zeta potential of M13/nLNP was −23.3 ± 0.9 mV in deionized water (n = 3); (**E**) representative high-performance liquid chromatography (HPLC) profile quantifying M13 in total M13/nLNP suspension (red), free M13 in filtered-through solution (blue), and the loading efficacy of M13 in nLNP ((n = 3); (**F**) M13/nLNP drug release profile in the ex vivo culture medium (pH~7.4); (**G**) modeling of the M13 release from the nLNP matrix (Higuchi model, R^2^ = 0.9424); (**H**) modeling of the M13 release from nLNP matrix, from left to right: zero-order kinetics (R^2^ = 0.8227), first-order kinetics (R^2^ = 0.9166), Korsmeyer–Peppas (R^2^ = 0.9061), and Hixson–Crowell model (R^2^ = 0.896).

We previously reported ginger-derived nanoparticles (GDNPs) as spherical nanoparticles with lipid bilayer envelopes encircling an aqueous center. GDNPs present a slightly negative surface charge, as the major lipids that form the membrane include phosphatidic acid (PA, negatively charged), monogalactosyldiacylglycerol (MGDG, neutral), and digalactosyldiacylglycerol (DGDG, neutral) [14,18]. GDNPs carry many ginger-derived biomolecules (e.g., proteins, microRNAs, and lipids) and secondary metabolites (e.g., gingerols and shogaols). As the concentrations of these molecules vary from batch to batch, the stability of GDNPs may be inconsistent. In an effort to develop these nanoparticles toward a more well-defined drug delivery system, we herein extracted the lipids from GDNPs using a liquid–liquid extraction method [15], used a thin-film hydration method to reassemble the lipids, and loaded the drug candidate, M13, into the generated nanoparticles. Atomic force microscopy (AFM) visualization showed that the M13-loaded reassembled nLNP (M13/nLNP) are spherical (Figure 1A) and have a hydrated average size of about 183 nm, a PDI of 0.196, and average zeta potential of −18.3 ± 0.5 mV (Figure 1B,C) under physiologically relevant conditions (in PBS). The average zeta potential of M13/nLNP in ultrapure water was measured as −23.3 ± 0.9 mV (Figure 1D), indicating a negatively charged particle surface.

We next measured the loading efficiency of M13 in nLNP by testing the free M13 concentration outside the nLNP (found in the filtered-through solution after centrifugation) versus the concentration of total M13 in the M13/nLNP suspension. A typical HPLC chromatograph of the free M13 solution versus total M13 is shown in Figure 1E (left). As presented in Figure 1E (right), data from three batches demonstrated that the M13/nLNP could achieve an M13 loading efficiency of up to 75.3 ± 1.7% at a 0.1 mg/mL lipid concentration.

We also tested the M13 drug release profile in the ex vivo culture medium by dialysis device (Figure 1F). We fitted the observed concentration-time data with mathematical models (zero-order, first-order, Higuchi, Korsmeyer–Peppas, and Hixson–Crowell models) [19]. As shown in Figure 1G, the drug release of M13 appeared to be the best fit with Higuchi’s square root model (R^2^ = 0.9424). The zero-order kinetic model, which indicates a concentration-independent Fickian diffusion, was fitted with a lower correlation coefficient (R^2^ = 0.8227). These results suggest that the mechanism through which M13 is released from nLNP is similar to that of a drug being released from control-released liposomes [20].

### 3.2. M13/nLNP Changes Ex Vivo Cultured Gut Microbiota Composition

**Figure 2:**
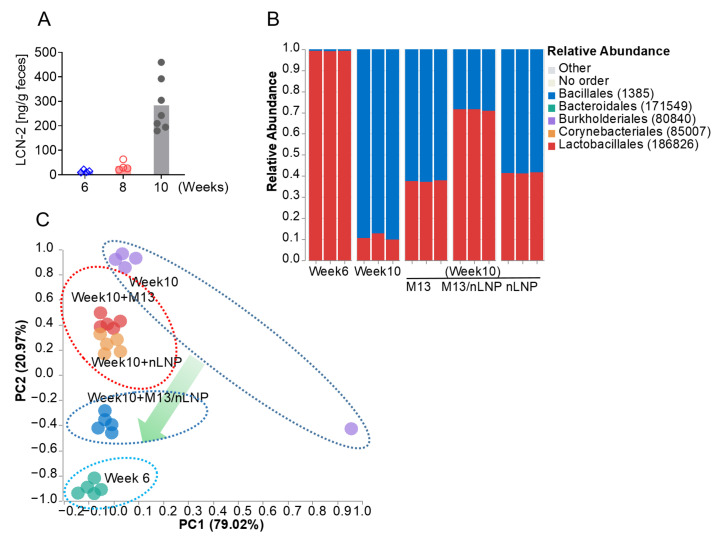
The changes in the ex vivo-cultured microbiota composition. (**A**) Fecal lipocalin-2 (LCN-2) levels of 6-, 8-, and 10-week-old IL-10 KO mice (n = 7); (**B**) taxonomic summary at the order level for ex vivo-cultured fecal microbiota cultured with medium spiked with PBS, M13, nLNP, or M13/nLNP (n = 3). (**C**) PCoA of microbiota composition (at the order level) after ex vivo culture (n = 5).

Fecal microbiota transfer (FMT) is the most widely used method to modulate gut microbiota, but this technique carries a high risk of infection to the recipient [21,22]. Here, we took a different approach to eliminate such infection risks. We first examined the capability of M13/nLNP to modulate the ex vivo cultured gut microbiota. We collected feces from 6- and 10-week-old IL-10 KO mice. As shown in Figure 2A, the fecal LCN-2 level was normal in 6-week-old mice but elevated in 10-week-old mice, suggesting that after 4 weeks of accommodation (from 6 weeks old), the mice developed mild inflammation. We then isolated microbiota from 10-week-old mice and ex vivo cultured the inflamed microbiota with M13/nLNP, PBS, free M13, or empty nLNP. A parallel cultured 6-week-old microbiota was used as a control. After 24 h of incubation, we tested the microbiota compositions of the different groups. As shown in Figure 2B, week 6 microbiota showed a dominant ratio of lactobacillales (red), whereas week 10 microbiota had a higher percentage of bacillales (blue) than lactobacillales. The M13/nLNP-cultured week 10 microbiota demonstrated the most potent ability to decrease the ratio of bacillales and increase the ratio of lactobacillales compared to free M13 and empty nLNP. In our principal coordinate analysis (PCoA; Figure 2C), the week 6 and PBS-treated week 10 microbiota compositions were separated by the largest distance. Interestingly, M13/nLNP treatment shifted the microbiota composition toward that of the 6 week (non-inflamed) microbiota. Various studies have identified lactobacillales as having probiotic activity [23,24]. Thus, the observed changes in the percentage of lactobacillales might indicate that M13/nLNP culturing has an anti-inflammatory effect.

### 3.3. Changes in Microbiota Composition Produce Distinctions in Secreted Metabolites

**Figure 3:**
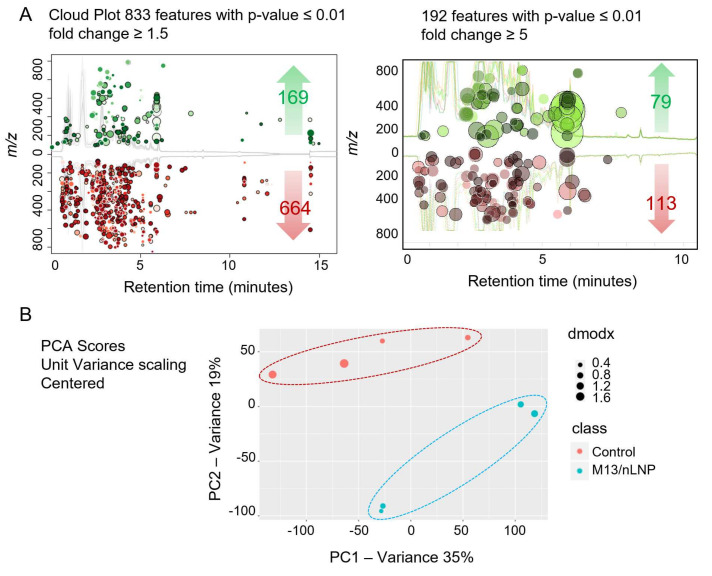
M13/nLNP alters ex vivo-cultured microbiota-secreted metabolites. (**A**) Cloud plot visualizes upregulated (green) and downregulated metabolites (red) in ex vivo M13/nLNP-cultured microbiota compared to the control group (PBS-cultured); (**B**) PCA analysis of M13/nLNP-cultured microbiota secreted metabolites (blue) and PBS-cultured microbiota secreted metabolites (control, red) (n = 4).

After finding that M13/nLNP could change the ex vivo-cultured microbiota composition, we next investigated whether this compositional change could yield different microbiota-secreted metabolites. Using a liquid chromatography-mass spectrometry (LC-MS)-based untargeted-metabolomics technique, we found that the metabolic profile was significantly changed after M13/nLNP-culture (Figure 3A,B). As shown in Figure 3A (left), a cloud plot demonstrated that more MS features were significantly downregulated (red; 664 of 833; 1.5-fold change) than upregulated (green; 169 of 833; 1.5-fold change) after treatment with M13/nLNP for 24 h. Further, 192 (out of 833) metabolites were significantly altered with a fold change larger than five (Figure 3A, right), with 79 (out of 192) metabolites upregulated (green) and 113 (out of 192) downregulated (red). Principal component analysis (PCA) demonstrated the presence of clear edges between the M13/nLNP-cultured microbiota-secreted metabolites (blue) and PBS-cultured microbiota-secreted metabolites (control, red), confirming that these groups could be easily discriminated by their respective microbiota-secreted metabolite profiles (Figure 3B).

### 3.4. Altered Microbial Metabolites Exert Anti-Inflammatory Effects In Vitro

Given our observation that M13/nLNP culturing increased the composition of possible probiotic lactobacillales and consequently modulated secreted metabolites, we next tested if the changes in the composition of secreted metabolites had an anti-inflammatory effect on the NF-κβ signal transduction pathway. NF-κβ is a central regulator of innate immunity and is believed to be associated with the development of ulcerative disease [25,26].

**Figure 4:**
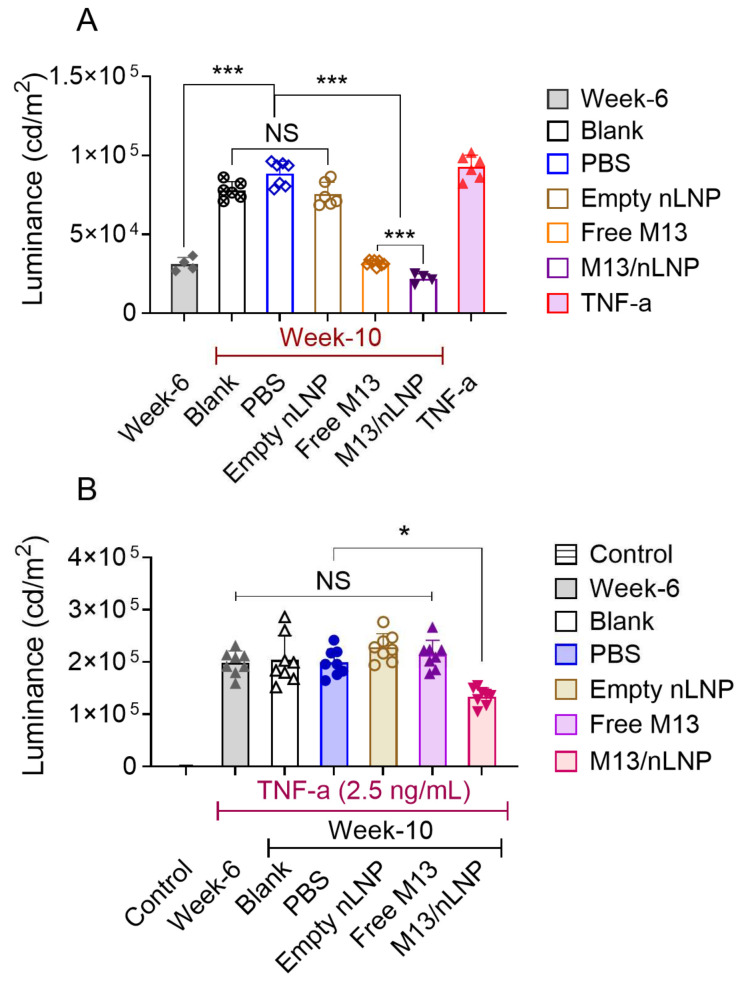
The effects of M13/nLNP-incubated microbiota-secreted metabolites on NF-κβ reporter cells. (**A**) Activation of NF-κβ signal transduction by the different groups of metabolites (n = 4–6; *** *p* < 0.001); (**B**) ability of different groups of metabolites to inhibit the ability of TNF-α to activate NF-κβ reporter cells (n = 8; * *p* < 0.05; NS—non-significant).

We first tested whether metabolites secreted by the non-inflamed (week-6) and the inflamed (week 10) fecal microbiota could induce activation of NF-κβ signaling. Indeed, we found that the week 10 microbiota (with or without PBS, or empty nLNP cultured)-secreted metabolites activated NF-κβ signal transduction in reporter cells, as did recombinant TNF-α (10 ng/mL) (Figure 4A). Free M13 or M13/nLNP-cultured week 10 microbial metabolites failed to activate NF-κβ signal transduction in the reporter cells, suggesting that the metabolite compositional change that occurred in the presence of M13 did not trigger NF-κβ signal transduction-associated inflammation. We further tested whether the microbiota-secreted metabolites could inhibit the TNF-α-induced stimulation of NK-kβ. Interestingly, only M13/nLNP-cultured microbiota-secreted metabolites inhibited the TNF-α-induced stimulation of NF-κβ signaling (Figure 4B). These results indicate that microbiota-derived metabolites from the M13/nLNP-treated group exerted an anti-inflammatory effect via the NF-κβ signal transduction pathway.

### 3.5. Metabolites Derived from M13/nLNP Cultured Microbiota Prevented the Development of UC

**Figure 5:**
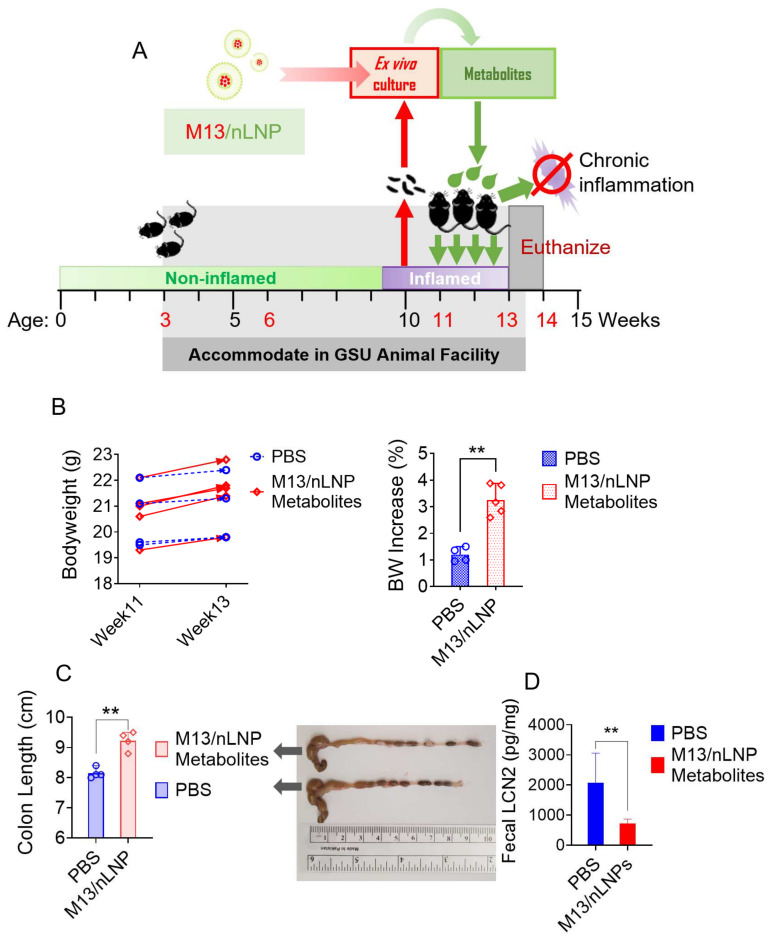
Effects of M13/nLNP-modified microbial metabolites on the UC progression of IL-10 KO mice. (**A**) Schematic diagram showing feces collection, ex vivo culture, and in vivo treatment; (**B**) bodyweight changes in control mice (PBS only; blue) and mice treated for 14 days with microbial metabolites from M13/nLNP-cultured group (red). (n = 4 or 5; ** *p* < 0.01); (**C**) colon lengths (n = 4; ** *p* < 0.01). (**D**) Fecal LCN-2 concentrations at week 13 (n = 4; ** *p* < 0.01).

Having demonstrated that microbial metabolites obtained following M13 culturing effectively inhibit NF-κβ signal transduction in vitro, we tested whether these metabolites could prevent the progression of UC in the in vivo transgenic mouse model. IL-10 KO mouse feces were monitored for their LCN-2 concentrations by ELISA. The 10-week-old mice presented an elevated concentration of LCN-2 (~300 ng/g) compared to the 6-week-old mice (<30 ng/g). During weeks 11 to 13, mice were given a once-daily oral dose of microbial metabolites from the M13/nLNP ex vivo-cultured group (isolated from 2 mL of cultured microbiota) by gavage (Figure 5A). The control group was provided with PBS. During the treatment period, the M13-nLNP microbiota-secreted metabolite group gained ~3% body weight, whereas the control group gained only ~1% body weight (Figure 5B), indicating that M13/nLNP microbiota-derived metabolites might have a beneficial effect on IL-10 KO mice. Measurements of colon length (Figure 5C) also showed a significantly longer average colon length in the M13-nLNP microbiota-secreted metabolite group than in the PBS control group, indicating that these metabolites could have a UC-preventing effect. A significant reduction in the fecal LCN-2 level in the M13/nLNP-cultured microbial metabolite group (Figure 5D) supported the notion that this treatment prevented the disease development of UC.

**Figure 6:**
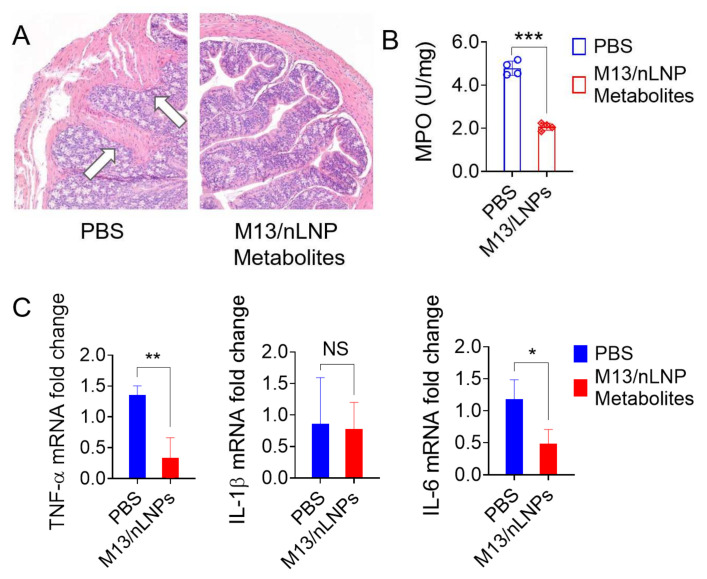
Histological analysis, MPO level, and pro-inflammatory mRNA levels of M13/nLNP-modified microbial metabolites during the UC progression of IL-10 KO mice. (**A**) H&E staining (white arrows indicate inflammatory cells in the lamina propria); (**B**) colonic MPO level; (**C**) measurement of mRNA levels of cytokines. (n = 4; *** *p* < 0.001, ** *p* < 0.01, * *p* < 0.05, NS—non-significant).

Histological analysis confirmed that intestinal mucosal ulceration was significantly decreased in mice treated with microbial metabolites from the M13/nLNP-cultured group (Figure 6A, right), whereas severe mucosal ulcerations were clearly present in the control group (Figure 6A, left, white arrows). We also found that microbial metabolites from the M13/nLNP-cultured group reduced colonic MPO activity (Figure 6B) and significantly decreased the mRNA expression levels of the pro-inflammatory cytokines, TNF-α and IL-6 (Figure 6C), compared to the PBS control. There was no between-group difference in the IL-1β mRNA level. The ability to decrease the mRNA expression levels of TNF-α and IL-6 in colon tissues may explain the efficacy of microbiota-secreted metabolites from the M13/nLNP-cultured group in preventing the deterioration of UC in IL-10 KO mice.

### 3.6. Oral Administration Microbiota-Secreted Metabolites Are Non-Toxic to IL-10 KO Mice

**Figure 7:**
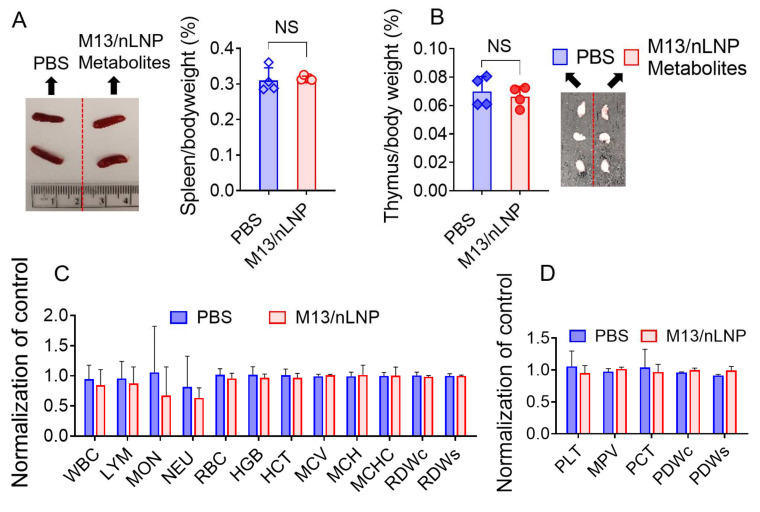
Safety evaluation for M13/nLNP-incubated microbiota-secreted metabolites in IL-10 KO mice. (**A**) Representative spleen picture and spleen-to-bodyweight ratio for M13/nLNP-incubated microbiota-secreted metabolite-treated and control mice; (**B**) thymus-to-bodyweight ratio and representative thymus pictures from the two groups; (**C**) blood was collected from the retro-orbital sinus, and hematological analyses were performed using an automatic hematology analyzer (VetScan HM5; Abaxis, CA, USA). The following hematologic parameters are shown: WBC—total white blood cells; LYM—lymphocytes; MON—monocytes; NEU—neutrophils; RBC—red blood cells; HGB—hemoglobin; HCT—hematocrit; MCV—mean corpuscular volume; MCH—hemoglobin amount per red blood cell; MCHC—mean corpuscular hemoglobin concentration; RDWc—red cell distribution width cv; RDWs—red cell distribution width sd; (n = 4). (**D**) PLT—platelets; MPV—mean platelet volume; PCT—procalcitonin; PDWc—platelet distribution width cv; PDWs—platelet distribution width sd. (n = 4).

Spleen and thymus weights are sensitive indicators of immunotoxicity (immune stimulation or depletion), stress, and physiologic perturbations [27,28]. The spleen- and thymus-to-bodyweight ratios reflect the immune response to drug treatment. Here, we found no significant difference in the spleen-to-bodyweight (Figure 7A) or thymus-to-bodyweight (Figure 7B) ratios of M13/nLNP-cultured microbiota metabolite-treated mice versus PBS control mice. We also compared the routine blood counts of M13/nLNP-cultured microbial metabolite-treated IL-10 KO mice with those of PBS-treated IL-10 KO control mice. The numbers for total white blood cells, lymphocytes, monocytes, neutrophils, red blood cells, hemoglobin, hematocrit, mean corpuscular volume, hemoglobin amount per red blood cell, mean corpuscular hemoglobin concentration, red cell distribution width, platelets, mean platelet volume, and platelet distribution width did not differ between the two groups (Figure 7C,D). Together, these data show that oral delivery of microbiota-secreted metabolites did not trigger an immune response in IL-10 KO mice, suggesting that such metabolite transfer might be safe for the host.

## 4. Discussion

The gut microbiota plays a crucial role in maintaining the hemostasis of the intestinal microenvironment via different pathways [29]. One well-recognized mechanism involves an interaction between chemical signaling messengers secreted from the microbiota and receptors present on the host cells [30,31,32,33]. These messengers include macromolecular immune modulators (e.g., bacterial flagellin) and small molecular metabolites (e.g., short-chain fatty acids and oligopeptides). A change in the microbiota composition often results in the secretion of a different set of small molecular microbial metabolites. As the microbial metabolites are small, they can easily pass through the mucus layer to interact with colonic cells and trigger immune responses [33,34,35,36]. Modifying gut microbiota-secreted metabolites may therefore have translational potential in modulating the progression of intestinal inflammation.

As UC is a chronic inflammatory disease, the IL-10 KO chronic inflammation model can better recapitulate the nature of UC than the DSS-induced acute colitis model [6,7,37]. During the progression of chronic inflammation in the colon, the mucus layer is not significantly compromised, and the permeability of the epithelial lining is not drastically modified, whereas both of these changes are seen in the acute colitis model. For this reason, many anti-inflammatory treatments developed based on the DSS-induced acute colitis model may fail to prevent the deterioration of UC in the clinic. Nanomedical treatments designed to target the enhanced permeability (EPR) effect exhibited by colonic epithelial tissues during inflammation or cancer, which have expanded in recent years, might only be suitable for the late stage of UC and have little effect in preventing the disease. Our strategy to tackle the progression of UC might offer an alternative and complementary approach for better managing this disease.

Studies have shown that lipids isolated from GDNPs can self-assemble to generate nano-sized natural LNP that can deliver drugs to modulate the gut microbiota and its secreted metabolites [38,39]. The high percentage of glycerol lipids (>50%) of the nanoplatform might contribute to the higher uptake rate of these nLNP, compared to synthetic polymeric nanoparticles, by the gut microbiota. The presence of galactosyl moieties in these glycerol lipids could enable them to target natural enteric bacteria; in several enteric bacteria, they may be utilized via the Leloir pathway for glycogenesis or the synthesis of mucopolysaccharides and glycoproteins [40,41]. Thus, nLNP-encapsulated drugs appear to be more capable than free drugs in modulating the composition and microbial metabolites of the microbiota.

The current study is a proof-of-principle study in which nLNP-delivered M13 demonstrated the ability to change the composition of an ex vivo cultured gut microbiota, which in turn secreted functionally altered microbiota-secreted metabolites. Both the nLNP-delivered drug and the drug-modified microbiota metabolites could be used as potential approaches for UC treatment. As the ex vivo-cultured gut microbiota could be amenable to mass production, it would be a more sustainable and cost-effective approach than direct administration of the nLNP-delivered drugs. Additionally, using the drug-modified microbiota metabolites might reduce the toxicity of the drug, as its concentration is largely reduced in the ex vivo culture process, and only the isolated microbial metabolites are delivered to the host.

One limitation of our study is that the alteration of the microbiota composition could be affected by the accumulation of secreted metabolites. As the concentration of metabolites increases, the growth of several species of bacteria might be inhibited. Thus, a dynamic flowing device must be developed to continuously eliminate the secreted metabolites from the medium so that the metabolites themselves will not affect how the nLNP-delivered drug changes the composition of cultured microbiota. Additionally, other key factors, such as the nLNP-encapsulated drug concentration, culture time, and anaerobic gas composition, can be further optimized. Although this proof-of-concept study demonstrates the anti-inflammatory effects of ex vivo modified metabolites, further investigations are required to characterize the specific bacteria that contribute to the anti-inflammatory metabolites and to identify anti-inflammatory metabolite structures.

## 5. Conclusions

Natural LNP-delivered M13 reshapes ex vivo-cultured inflamed microbiota and renders downstream compositional changes in microbiota-secreted metabolites. The altered microbial metabolites inhibit the ability of TNF-α to activate the NF-κβ pathway in NF-κβ reporter cells. Our findings collectively demonstrate that autologous metabolites transferred from colitogenic microbiota treated with anti-inflammatory drug-encapsulating lipid nanoparticles can prevent ulcerative colitis in a mouse model.

## Data Availability

The data underlying this article will be shared at reasonable request to the corresponding author.
